# Extracellular Vesicles Mediate Epimastigogenesis in *Trypanosoma cruzi*: Strain-Specific Dynamics and Temperature-Dependent Differentiation

**DOI:** 10.3390/life15060931

**Published:** 2025-06-09

**Authors:** Abel Sana, Izadora Volpato Rossi, Bruna Sabatke, Marcel Ivan Ramirez

**Affiliations:** 1 EVAHPI—Extracellular Vesicles and Host-Parasite Interactions Research Group, Laboratório de Biologia Celular, Instituto Carlos Chagas-Fiocruz, Curitiba 81350-010, Brazil; sanaabel@ufpr.br (A.S.); izadoravolpato@ufpr.br (I.V.R.); bruna.sabatke@ufpr.br (B.S.); 2 Programa de Pós-Graduação em Biologia Celular e Molecular, Universidade Federal do Paraná (UFPR), Curitiba 80060-000, Brazil; 3 Programa de Pós-Graduação em Microbiologia, Parasitologia e Patologia, Universidade Federal do Paraná (UFPR), Curitiba 80060-000, Brazil; 4 Laboratório de Biologia Celular, Instituto Carlos Chagas-Fiocruz, Curitiba 81350-010, Brazil

**Keywords:** *Trypanosoma cruzi* cell diferentiation, extacelular vesicles, epimastigogenese, parasite insect interaction

## Abstract

*Trypanosoma cruzi*, the causative agent of Chagas disease, undergoes epimastigogenesis—a critical differentiation step in which trypomastigotes transform into epimastigotes. While this process is essential for the parasite’s survival in its insect vector, the molecular mechanisms regulating it remain poorly understood. Here, we present the first evidence implicating extracellular vesicles (EVs) as enhancing mediators of epimastigogenesis. Using in vitro models with *T. cruzi* strains CL Brener and Dm28c, we demonstrate that EVs, membrane-bound vesicles, were shown to enhance differentiation in a strain-specific and temperature-dependent manner. We observed strain-specific EV release patterns: Dm28c produced more EVs at 24 h, whereas CL Brener peaked at 72 h. Additionally, we confirm that epimastigogenesis occurs exclusively at 28 °C after 72 h. These findings establish EVs as novel regulators of *T. cruzi* differentiation and suggest new insight into parasite development, highlighting potential targets for therapeutic intervention. The observed enhancement of differentiation in the presence of EVs indicates a potential role for these vesicles in this developmental process, although the underlying mechanisms remain undefined.

## 1. Introduction

*Trypanosoma cruzi*, the causative agent of Chagas disease, is a public health problem affecting between 8 and 12 million people worldwide [1]. This protozoan parasite belongs to the genus Trypanosoma and the family Trypanosomatidae [2], and is classified under the order Kinetoplastida due to the presence of a kinetoplast, a specialized mitochondrial DNA-containing structure [3]. Its life cycle is dixenous, requiring both a vertebrate host—such as humans or wild and domestic animals—and an invertebrate vector, specifically, hematophagous insects from the Triatominae subfamily [4].

Throughout its life cycle, T. cruzi transitions through four principal morphological stages: metacyclic trypomastigotes, amastigotes, bloodstream trypomastigotes, and epimastigotes [5]. Among these, trypomastigotes (metacyclic and bloodstream) are infective forms, whereas amastigotes and epimastigotes are responsible for replication [5,6]. Morphologically, these forms are distinguished by the position of the kinetoplast in relation to the nucleus—posterior in trypomastigotes and anterior in epimastigotes ([6])—and by differences in complement susceptibility [7].

Differentiation events are crucial for parasite survival and transmission, including the transformation of trypomastigotes into amastigotes and vice versa within the mammalian host, and the conversion of epimastigotes into metacyclic trypomastigotes in the insect vector [4,8]. Among these, epimastigogenesis, or the transformation of trypomastigotes into epimastigotes, has been relatively understudied. This process occurs naturally in the digestive tract of the vector, but can also be reproduced in vitro [4,9]. While some morphological and antigenic changes during epimastigogenesis have been described [8,10] the mechanisms of cellular communication involved remain unknown.

Extracellular vesicles (EVs) are membrane-bound particles secreted by cells, involved in intercellular communication through the transfer of proteins, lipids, and nucleic acids [11,12]. They are typically classified into exosomes and microvesicles based on their biogenesis. EVs have been implicated in parasite–parasite and parasite–host interactions, as shown in T. vaginalis, where EVs mediate communication between strains and enhance host cell adherence [13,14]. In T. cruzi, EVs have been reported to modulate the immune system, carrying mucins and TS/GP85 glycoproteins that trigger inflammation or inhibit complement activation [1,15]. Furthermore, EVs have been linked to metacyclogenesis, promoting the transformation of epimastigotes into infective metacyclic trypomastigotes [16]. In addition to these proteins, EVs from T. cruzi have been found to contain small RNAs and specific lipid profiles, which may contribute to vesicle stability, target specificity, and signalling capacity [1,16,17]. These components have been characterised in earlier studies, though their functional relevance during epimastigogenesis remains unclear [18,19].

Understanding the EV cargo during this stage could reveal molecular signatures or triggers that influence parasite development. Moreover, investigating the downstream signalling pathways activated by EVs could help identify new mechanisms of stage regulation [20].

Despite these advances, the potential role of EVs in epimastigogenesis has not been explored. In this study, we investigate this unexplored area using in vitro models and two *T. cruzi* strains, CL Brener and Dm28, to determine whether EVs influence the transformation of trypomastigotes into epimastigotes. We also evaluate the temperature dependency of the process and characterise the EV release profile during differentiation.

## 2. Methods

### 2.1. Parasite Cultivation

Cell culture-derived trypomastigotes (TCTs) of *T. cruzi* strains CL Brener and Dm28c were maintained through cycles of infection in Vero cells cultured in RPMI 1640 medium supplemented with 10% fetal bovine serum (FBS) at 37 °C, as described by Rossi et al. (2024) [21].

### 2.2. In Vitro Epimastigogenesis

TCTs were harvested from the supernatant of infected Vero cells after five days of infection and washed with serum-free LIT culture medium at 3000× *g* for five minutes. Then, 1 × 10^6^ TCTs/mL were incubated in the same culture medium at 28 °C and 37 °C to induce differentiation. The numbers of TCTs and differentiated forms were quantified using a Neubauer chamber after 24 and 72 h of differentiation.

### 2.3. Infection Assays

TCTs were cultured to induce epimastigogenesis as described above. Parasites after 72, 120, and 168 h of epimastigogenesis were harvested and washed with RPMI 1640 without FBS and used to infect Vero cells. For this, Vero cells were cultured on 13 mm coverslips in a 24-well plate at a density of 5 × 10^4^/well for 4 h at 37 degrees. After the incubation period, the cells were washed with RPMI 1640 without FBS and incubated in the presence of differentiated parasites (5 × 10^5^/mL) for 4 h. The cells were washed 3 times with RPMI 1640 and incubated with the same medium supplemented with FBS (5%) at 37 °C, for approximately 18 h. Cells were washed with 1× PBS, fixed with 4% paraformaldehyde for 20 min at room temperature, washed again with 1× PBS, and stained with Giemsa stain (diluted 1:10) for 12 min. The cells were visualised and quantified using an optical microscope at 1000-fold magnification to determine the number of infected cells and intracellular parasites. We counted at least 300 cells, from which we calculated the percentage of infected cells and the total number of intracellular parasites. Undifferentiated TCTs were used as a positive control and Epimastigotes as a negative control.

### 2.4. Isolation and Characterisation of Extracellular Vesicles

EVs were isolated from the supernatant of parasites undergoing epimastigogenesis using differential centrifugation. TCTs at a density of 2 × 106/mL were incubated in serum-free LIT medium at 28 °C. After 24 and 72 h, the samples were centrifuged at 3000× *g*, 10 °C, for five minutes to pellet the parasites. The supernatant was transferred to a new microtube and centrifuged at 4000× *g*, 4 °C, for 30 min to remove debris. The cell- and debris-free supernatant was centrifuged at 15,000× *g*, 4 °C, for two hours to isolate EVs ([22]). EVs were characterised for concentration and size using nanoparticle tracking analysis (NTA). It is important to note that while differential centrifugation was used to isolate EVs, this method may not completely eliminate the presence of parasite debris. To improve the specificity of EV isolation, future experiments will incorporate ultracentrifugation and/or density gradient separation methods.

### 2.5. Epimastigogenesis in the Presence of EVs

Epimastigogenesis was assessed in the presence of EVs previously isolated from the parasite supernatant after 24 and 72 h of differentiation, as described above. TCTs (5 × 10^5^/mL) were incubated in the presence of EVs (1 × 10^7^/mL) obtained at 24 and 72 h. After 72 h of incubation at 28 °C, the number of epimastigotes was assessed by morphological criteria.

### 2.6. Morphological Analysis

The morphology of parasites in each experimental condition was analysed after 72 h of epimastigogenesis. Parasites at a density of 2.5 × 10^7^/mL were fixed in methanol for two minutes and stained with Giemsa dye (diluted 1:10) for 12 min. An aliquot of 50 µL was visualised and quantified using an optical microscope at 1000-fold magnification. The position of the kinetoplast in relation to the nucleus was used as a criterion to determine the percentage of trypomastigotes, parasites undergoing differentiation, and epimastigotes.

Parasite morphology was also examined under a fluorescence microscope. After 72 h of epimastigogenesis, the parasites (5 × 10^5^/mL) were labelled with carboxyfluorescein succinimidyl ester (CFSE) and incubated for 15 min at room temperature. Then, the parasites were washed twice in 1× PBS by centrifuging the samples at 3000× *g* for five minutes, fixed in methanol for two minutes, and examined using a fluorescence microscope.

### 2.7. Statistical Analysis

All experiments were performed in triplicate. Data were analysed using one-way ANOVA followed by Tukey’s post hoc test for multiple comparisons. Results were considered statistically significant when *p* < 0.05. Statistical analyses were conducted using GraphPad Prism version 6.1.

## 3. Results

### 3.1. Epimastigogenesis: A Temperature-Driven Transformation

**Figure 1:**
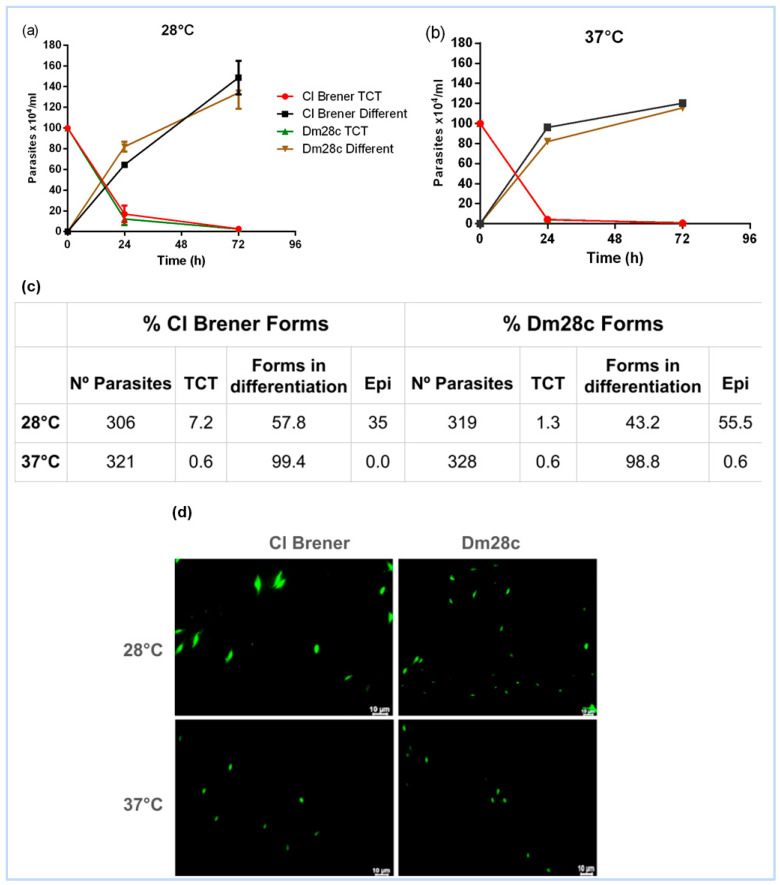
Determination of CL Brener and Dm28c epimastigogenesis after 72 h of incubation at 28 °C and 37 °C. (**a**,**b**) Quantification of TCTs and differentiated forms using a Neubauer chamber at 24 and 72 h. (**c**) Giemsa staining shows the differentiation of *T. cruzi* into various forms after 72 h. (**d**) Fluorescence microscopy illustrating parasite morphology at 28 °C and 37 °C.

We analysed the in vitro epimastigogenesis of cell culture-derived trypomastigotes (TCTs) from *T. cruzi* strains CL Brener and Dm28c at 28 °C and 37 °C. Both strains exhibited morphological changes at both temperatures after 72 h (Figure 1a,b). However, differentiation into epimastigotes occurred exclusively at 28 °C (Figure 1c,d). The Dm28c strain demonstrated higher efficiency (55.5%) compared to CL Brener (35%) (Figure 1c).

### 3.2. Evaluation of the Invasive Capacity of T. cruzi CL Brener and Dm28c After Epimastigogenesis

We assessed the invasive capacity of *T. cruzi* strains CL Brener and Dm28c after 72 h of in vitro epimastigogenesis at 28 °C. Trypomastigote cell culture-derived forms (TCTs), which are naturally invasive, were used as the starting point for differentiation. Parasites were maintained under differentiation conditions for 72 h, after which the resulting population—comprising a mixture of residual TCTs, intermediate forms, and epimastigotes—was used in infection assays.

**Figure 2:**
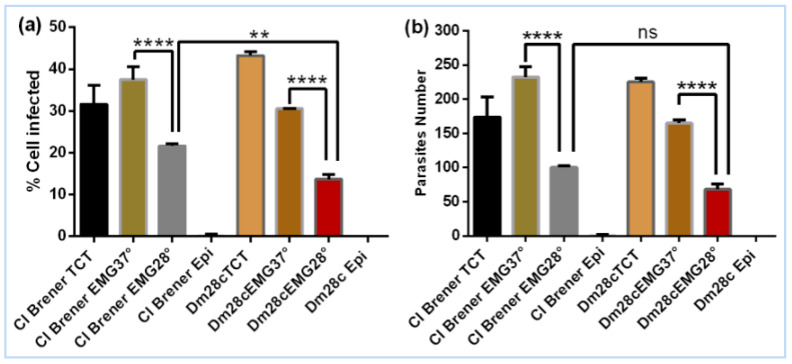
Evaluation of the invasive capacity of CL Brener and Dm28c after 72 h of differentiation at 28 °C and 37 °C. (**a**) Percentage of infected cells. (**b**) Average number of intracellular parasites. **** *p* < 0.0001, ** *p* < 0.01, ns—not significant.

The results showed that after 72 h of epimastigogenesis at 28 degrees, both CL Brener and Dm28c are still able to invade Vero cells, as already shown in Dm28c [10]. The number of infected cells was higher in CL Brener (21.6%) compared to Dm28c (13.6%) (Figure 2a); however, there was no significant difference in the number of intracellular parasites (Figure 2b). A significant difference was observed in the number of infected cells and in the number of intracellular parasites in both strains when comparing the invasion of parasites differentiated at 37 degrees to parasites differentiated at 28 degrees (Figure 2a,b). The reduction in invasive capacity at 28 degrees suggests that it is due to the high number of epimastigote forms in this condition compared to 37 degrees, as shown in Figure 1c.

**Figure 3:**
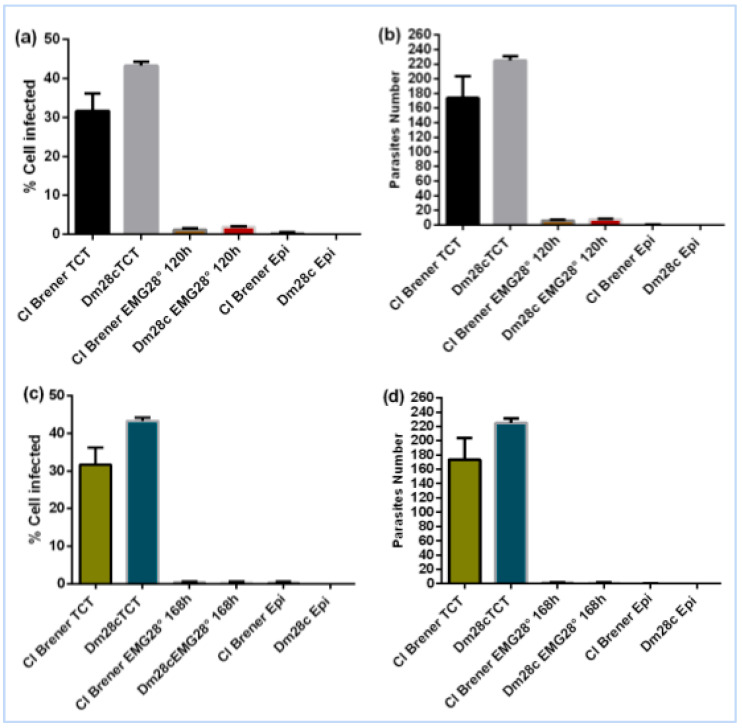
Evaluation of the invasive capacity of CL Brener and Dm28c after 120 h and 168 h of differentiation at 28 °C. (**a**) Percentage of infected cells. (**b**) Number of intracellular parasites. (**c**) Percentage of cells infected (**c**) with parasites after 168 h of differentiation; (**d**) Number of intracellular parasites after 168 h of differentiation.

This observation supports the notion that the infectivity of *T. cruzi* is closely linked to its developmental stage, with TCTs being the most competent, intermediate forms showing reduced capacity, and mature epimastigotes being non-infective. To confirm the complete loss of invasive potential, we extended the epimastigogenesis process to 120 and 168 h, at which point the cultures predominantly consisted of fully differentiated epimastigotes. As expected, no significant invasion was observed at these stages, confirming that mature epimastigotes are not capable of invading eukaryotic cells (Figure 3). After 120 h of epimastigogenesis, the invasive capacity of both CL Brener and Dm28c drops dramatically (Figure 3a,b) and reaches zero level after 168 h (Figure 3c,d). These findings reinforce the importance of developmental stage in the infective behaviour of *T. cruzi* and provide a robust experimental framework for studying stage-specific functions and vesicle-mediated modulation during parasite differentiation.

### 3.3. Extracellular Vesicles: Key Players in Epimastigogenesis

**Figure 4:**
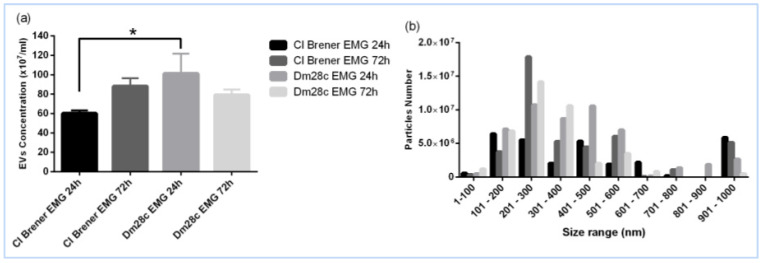
NTA of EVs isolated from supernatant after 24 and 72 h of epimastigogenesis (EMG). (**a**) Particle concentration. (**b**) Particle size distribution. Asterisks indicate statistical differences between groups. * *p* < 0.01.

To assess EV release during in vitro epimastigogenesis (EMG), supernatants from parasites incubated at 28 °C were collected at 24 (EMG 24 h) and 72 h (EMG 72 h). EVs were isolated using differential centrifugation, and their concentration and size were evaluated via nanoparticle tracking analysis (NTA). The Dm28c strain exhibited higher EV production at 24 h, whereas CL Brener reached its peak at 72 h. EV sizes varied, with CL Brener producing a significant fraction measuring 901–1000 nm (Figure 4a,b).

### 3.4. Unveiling the Role of EVs in Parasite Differentiation

**Figure 5:**
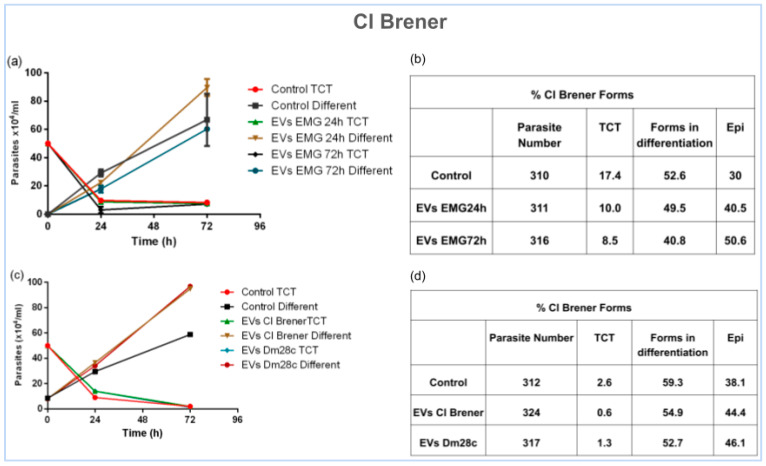
Effect of EVs on CL Brener epimastigogenesis after 72 h of incubation. (**a**,**c**) Quantification of TCTs and differentiated forms using a Neubauer chamber. (**b**,**d**) Giemsa staining showing the differentiation of *T. cruzi* forms. Control TCT—trypomastigote forms in the condition not treated with EVs. Control Different—non-trypomastigote forms in the condition not treated with EVs.

To evaluate the effect of EVs on epimastigogenesis, TCTs from CL Brener were treated with EVs (1 × 10^7^/mL) isolated from the same strain after 24 and 72 h of differentiation. The addition of EVs significantly enhanced the differentiation rate. Epimastigote formation increased to 40.5% with EVs from 24 h cultures and 50.6% with EVs from 72 h cultures, compared to 30% in controls (Figure 5a,b). Treatment of CL Brener with Dm28c EVs isolated after 72 h of differentiation showed a small increase in the amount of epimastigotes compared to the control (Figure 5c,d).

Quantitative analysis confirmed that the addition of EVs significantly enhanced the differentiation of trypomastigotes into epimastigotes in a strain- and time-dependent manner. In the presence of EVs isolated from supernatant after 24 and 72 h of epimastigogenesis, the number of epimastigotes increased approximately 35% and 68.6%, respectively, compared to the control (Figure 5b). Treatment of CL Brener with Dm28c EVs increased the number of epimastigotes by approximately 21% compared to the control (Figure 5d). These findings suggest a mechanism of EV-mediated cell communication in the differentiation of *T. cruzi* trypomastigotes into epimastigotes.

## 4. Discussion

### 4.1. Temperature as a Key Driver of Epimastigogenesis

The transition from trypomastigote to epimastigote is a temperature-sensitive process, reflecting the environmental shift that T. cruzi experiences upon entering the insect vector. Consistent with previous studies ([9,23]), our results confirm that in vitro epimastigogenesis occurs exclusively at 28 °C after 72 h, although early morphological changes can be observed at both 28 °C and 37 °C in CL Brener and Dm28c strains. This highlights temperature as a critical cue for initiating the differentiation process, likely mimicking the conditions of the insect midgut. In summary, epimastigogenesis in T. cruzi is temperature-driven and marked by a gradual loss of invasiveness, with extracellular vesicles enhancing differentiation in a strain-specific manner.

This observation is consistent with previous studies showing that morphological transformation is tightly linked to temperature variation and parasite stage transition, with extracellular vesicles enhancing differentiation in a strain-specific manner

Our findings demonstrate a clear influence of temperature on the initiation and progression of epimastigogenesis in T. cruzi, particularly in association with extracellular vesicle release. While this study focused on temperature as a primary trigger, we acknowledge that additional environmental factors such as pH ([24]), nutrient availability ([25]), and microbiota composition ([26]) may also influence epimastigogenesis and should be considered in future studies using more complex in vitro or in vivo models. These factors more closely mimic the physiological conditions encountered by the parasite within the insect vector and could reveal additional regulatory cues.

### 4.2. Loss of Invasive Capacity During Epimastigogenesis

Our findings demonstrate that T. cruzi progressively loses its ability to invade mammalian cells during epimastigogenesis. While parasites after 72 h of differentiation at 28 °C still retain partial infectivity, a sharp decline is observed after 120 h, culminating in complete loss of invasion capacity at 168 h. This correlates with the predominance of fully differentiated epimastigotes, which are naturally non-infective ([9,10]). These observations reinforce that invasive potential is tightly linked to the trypomastigote stage and suggest that epimastigogenesis involves profound functional remodelling, including changes in surface composition, signalling pathways, and cytoskeletal architecture ([7,23]). Understanding these mechanisms may provide new avenues for interfering with parasite development and transmission.

### 4.3. Extracellular Vesicles: Mediators of Differentiation

The role of EVs in the differentiation of *T. cruzi* was previously described in the context of metacyclogenesis, where EVs were shown to promote the transformation of epimastigotes into metacyclic trypomastigotes [16]. However, our study is the first to demonstrate a role for EVs in epimastigogenesis, revealing that these vesicles are not only released during differentiation, but actively enhance the transformation process. Importantly, EVs derived from 72 h cultures had a greater effect than those from 24 h cultures, suggesting maturation or accumulation of pro-differentiation signals over time.

The release of EVs exhibited strain-specific patterns: CL Brener released more EVs after 72 h, whereas Dm28c showed greater release within the first 24 h. Based on this, we hypothesise that: (1) Both strains respond differently to the sudden temperature drop (from 37 °C to 28 °C); (2) Dm28c is more sensitive to temperature changes, resulting in an early burst of EV production, which stabilises over time. CL Brener exhibits a delayed but sustained EV response, possibly requiring more time for differentiation signalling; (3) the balance between EV release and uptake differs between strains. Further studies are needed to validate these hypotheses and elucidate the mechanisms governing these differences.

### 4.4. Heterogeneity of EVs in Epimastigogenesis

Nanoparticle tracking analysis (NTA) revealed a broad size distribution and heterogeneity in EV sizes released during epimastigogenesis in both strains. The diversity of EV sizes suggests the involvement of multiple vesicle subtypes including small and large vesicles in this process. The observed differences in EV release patterns between strains indicate strain-specific regulatory mechanisms, reflecting distinct mechanisms of cargo delivery or signalling strength, warranting further investigation into their biological significance. This size variation likely reflects the presence of exosome-like vesicles (also referred to as small EVs) and microvesicle-like vesicles (also referred to as large EVs), indicating functional and biogenetic heterogeneity in the EV population released during epimastigogenesis.

### 4.5. Pioneering Evidence of EVs in Epimastigogenesis

This study is the first to demonstrate the involvement of EVs in epimastigogenesis, advancing our understanding of this critical differentiation process in *T. cruzi*. The findings suggest that EVs facilitate parasite communication and adaptation, potentially playing a similar role in vivo within the insect vector. In future studies, we intend to focus on the role of EVs in the development of epimastigogenesis in insect vectors, as well as on the evaluation of aspects of parasite survival and migration within the insect host.

By establishing a direct link between EVs and epimastigogenesis, this work opens new avenues for exploring EV-based therapeutic strategies aimed at disrupting key stages of the parasite’s life cycle.

These findings may have in vivo relevance, particularly within the insect vector, where EVs could influence parasite density, migration, or susceptibility to host factors. Future investigations will explore: the molecular composition of EVs released during epimastigogenesis; EV uptake and the signalling pathways activated by them; the role of EVs in insect colonisation and vector competence; whether EVs derived from host cells also modulate differentiation.

## 5. Conclusions

This study provides the first experimental evidence that EVs act as modulators of epimastigogenesis in *T. cruzi*. We demonstrate that EVs released during in vitro differentiation significantly enhance the transformation of trypomastigotes into epimastigotes, and that this effect is both strain-specific and temperature-dependent. These findings suggest that EVs are associated with epimastigogenesis, possibly contributing to communication and coordination during parasite development, although the precise mechanisms remain to be elucidated.

Furthermore, investigating the signalling mechanisms activated by EVs would strengthen this study’s conclusions. Future research focused on the molecular content of EVs and their functional pathways will be crucial to better understand their role in *T. cruzi* biology.

Our findings confirm that epimastigogenesis occurs exclusively at 28 °C, mimicking the insect vector environment, and that EV production differs temporally between strains, with Dm28c releasing more EVs at 24 h and CL Brener peaking at 72 h. The EV populations were heterogeneous in size, suggesting the involvement of multiple vesicle subtypes, potentially including both exosomes and microvesicles.

These results introduce a new conceptual layer to the regulation of *T. cruzi* development, suggesting that parasite-derived EVs may serve as intercellular signals that coordinate differentiation events during vector colonisation. In doing so, this work sets the stage for future studies exploring the molecular cargo of EVs, their functional impact in vivo, and their potential exploitation as diagnostic markers or therapeutic targets to disrupt the *T. cruzi* life cycle. These insights may eventually support the development of EV-based diagnostic markers or therapeutic interventions targeting differentiation pathways essential for *T. cruzi* survival.

In summary, the identification of EVs as key players in epimastigogenesis enhances our understanding of parasite biology and offers promising avenues for novel intervention strategies in Chagas disease control.

## Data Availability

The original contributions presented in this study are included in the article. Further inquiries can be directed to the corresponding author.

## References

[1] Torrecilhas A.C., Soares R.P., Schenkman S., Fernández-Prada C., Olivier M. (2020). Extracellular vesicles in trypanosomatids: Host cell communication. Front. Cell. Infect. Microbiol..

[2] Melo R.F.P., Guarneri A.A., Silber A.M. (2020). The influence of environmental cues on the development of *Trypanosoma cruzi* in Triatominae vector. Front. Cell. Infect. Microbiol..

[3] Cavalcanti D.P., de Souza W. (2018). The kinetoplast of trypanosomatids: From early studies of electron microscopy to recent advances in atomic force microscopy. Scanning.

[4] Silva B., Gregorio J., Contreras U.L., Graterol R., Aguilera N., Navarro M.C., Contreras A., de Lima R. (2008). *Trypanosoma cruzi*: Epimastigogenesis en condiciones axénicas. Cambios morfológicos, peptídicos, glicopeptídicos y antigénicos. Parasitol. Acta Científica Venez..

[5] Ferreira A.Z.L., de Araújo C.N., Cardoso I.C.C., de Souza Mangabeira K.S., Rocha A.P., Charneau S., Santana J.M., Motta F.N., Bastos I.M.D. (2023). Metacyclogenesis as the starting point of Chagas disease. Int. J. Mol. Sci..

[6] Rey L. (2008). Parasitologia: Parasitas e Doenças Parasitárias do Homem nos Trópicos Ocidentais.

[7] Cestari I., Evans-Osses I., Schlapbach L.J., de Messias-Reason I., Ramirez M.I. (2013). Mechanisms of complement lectin pathway activation and resistance by trypanosomatid parasites. Mol. Immunol..

[8] De Lima A.R., Aparicio A., Berrocal A., Navarro M.C., Graterol D., Contreras V.T. (2007). Epimastigogénesis de *Trypanosoma cruzi* en medio axénico: Cambios peptídicos, glicopeptídicos y enzimáticos. Salus.

[9] Graterol D., Arteaga R.Y., Navarro M.C., Domínguez M., de Lima A.R., Contreras V.T. (2013). El estadio amastigota precede la evolución del epimastigota durante la epimastigogénesis in vitro de *Trypanosoma cruzi*. Rev. Soc. Venez. Microbiol..

[10] Kessler R.L., Contreras V.T., Marliére N.P., Guarneri A.A., Silva L.H.V., Mazzarotto G.A.C.A., Batista M., Soccol V.T., Krieger M.A., Probst C.M. (2017). Recently differentiated epimastigotes from *Trypanosoma cruzi* are infective to the mammalian host. Mol. Microbiol..

[11] Hill A.F. (2019). Extracellular vesicles and neurodegenerative diseases. J. Neurosci..

[12] Urabe F., Kosaka N., Ito K., Kimura T., Egawa S., Ochiya T. (2020). Extracellular vesicles as biomarkers and therapeutic targets for cancer. Am. J. Physiol..

[13] Salas N., Blasco Pedreros M., Dos Santos Melo T., Maguire V.G., Sha J., Wohlschlegel J.A., Pereira-Neves A., de Miguel N. (2023). Role of cytoneme structures and extracellular vesicles in Trichomonas vaginalis parasite-parasite communication. eLife.

[14] Kochanowsky J.A., Mira P.M., Elikaee S., Muratore K., Rai A.K., Riestra A.M., Johnson P.J. (2024). Trichomonas vaginalis extracellular vesicles up-regulate and directly transfer adherence factors promoting host cell colonisation. Proc. Natl. Acad. Sci. USA.

[15] Cestari I., Ansa-Addo E., Deolindo P., Inal J.M., Ramirez M.I. (2012). *Trypanosoma cruzi* immune evasion mediated by host cell-derived microvesicles. J. Immunol..

[16] Garcia-Silva M.R., das Neves R.F., Cabrera-Cabrera F., Sanguinetti J., Medeiros L.C., Robello C., Naya H., Fernandez-Calero T., Souto-Padron T., de Souza W. (2014). Extracellular vesicles shed by *Trypanosoma cruzi* are linked to small RNA pathways, life cycle regulation, and susceptibility to infection of mammalian cells. Parasitol. Res..

[17] Cortes-Serra N., Gualdron-Lopez M., Pinazo M.J., Torrecilhas A.C., Fernandez-Becerra C. (2022). Extracellular Vesicles in *Trypanosoma cruzi* Infection: Immunomodulatory Effects and Future Perspectives as Potential Control Tools against Chagas Disease. J. Immunol. Res..

[18] Reis-Cunha J.L., Baptista R.P., Rodrigues-Luiz G.F., Coqueiro-Dos-Santos A., Valdivia H.O., de Almeida L.V., Cardoso M.S., D’Ávila D.A., Dias F.H.C., Fujiwara R.T. (2018). Whole genome sequencing of Trypanosoma cruzi field isolates reveals extensive genomic variability and complex aneuploidy patterns within TcII DTU. BMC Genom..

[19] Cronemberger-Andrade A., Xander P., Soares R.P., Pessoa N.L., Campos M.A., Ellis C.C., Grajeda B., Ofir-Birin Y., Almeida I.C., Regev-Rudzki N. (2020). Trypanosoma cruzi-Infected Human Macrophages Shed Proinflammatory Extracellular Vesicles That Enhance Host-Cell Invasion via Toll-Like Receptor 2. Front. Cell Infect. Microbiol..

[20] Retana Moreira L., Prescilla-Ledezma A., Cornet-Gomez A., Linares F., Jódar-Reyes A.B., Fernandez J., Ibarrola Vannucci A.K., De Pablos L.M., Osuna A. (2021). Biophysical and Biochemical Comparison of Extracellular Vesicles Produced by Infective and Non-Infective Stages of *Trypanosoma cruzi*. Int. J. Mol. Sci..

[21] Rossi I.V., de Almeida R.F., Sabatke B., de Godoy L.M.F., Ramirez M.I. (2024). *Trypanosoma cruzi* interaction with host tissues modulate the composition of large extracellular vesicles. Sci. Rep..

[22] Sana A., Rossi I.V., Sabatke B., Bonato L.B., Medeiros L.C.S., Ramirez M.I. (2023). An Improved Method to Enrich Large Extracellular Vesicles Derived from Giardia intestinalis through Differential Centrifugation. Life.

[23] Lander N., Chiurillo M.A., Docampo R. (2021). Signalling pathways involved in environmental sensing in *Trypanosoma cruzi*. Mol. Microbiol..

[24] Lander N., Chiurillo M.A., Bertolini M.S., Storey M., Vercesi A.E., Docampo R. (2018). Calcium-sensitive pyruvate dehydrogenase phosphatase is required for energy metabolism, growth, differentiation, and infectivity of *Trypanosoma cruzi*. J. Biol. Chem..

[25] Barisón M.J., Rapado L.N., Merino E.F., Furusho Pral E.M., Mantilla B.S., Marchese L., Nowicki C., Silber A.M., Cassera M.B. (2017). Metabolomic profiling reveals a finely tuned, starvation-induced metabolic switch in *Trypanosoma cruzi* epimastigotes. J. Biol. Chem..

[26] Castro D.P., Moraes C.S., Gonzalez M.S., Ratcliffe N.A., Azambuja P., Garcia E.S. (2012). *Trypanosoma cruzi* immune response modulation decreases microbiota in Rhodnius prolixus gut and is crucial for parasite survival and development. PLoS ONE.

